# Targeted Metabolomics With a Chemometric Study of Oxygenated Heterocyclic Aglycones as a Tool for Preliminary Authenticity Assessment of Orange and Grapefruit Juices

**DOI:** 10.3389/fnut.2022.897982

**Published:** 2022-05-23

**Authors:** Leng Han, Yujiao Cheng, Tenghui Zhang, Qi Zhou, Wanchao Zhang, Yongan Li, Guijie Li

**Affiliations:** ^1^Citrus Research Institute, National Citrus Engineering Research Center, Southwest University, Chongqing, China; ^2^Chengdu Centre Testing International Group Co., Ltd., Chengdu, China; ^3^Chongqing Institute of Medicinal Plant Cultivation, Chongqing, China; ^4^Administration of Agriculture and Rural Affairs of the Dongpo District, Meishan, China

**Keywords:** citrus, oxygenated heterocyclic compounds, profiling, variable reduction, partial least squares discriminant analysis, labeling, adulteration

## Abstract

Profiles of citrus juice oxygenated heterocyclic aglycones (OHAs), which are notable marker secondary metabolites, were used to assess the authenticity of sweet orange and grapefruit juices in situations where mandarin and pomelo juices might be adulterants. Thirty-nine known OHAs, including 10 methoxyflavones, 13 coumarins, and 16 furanocoumarins, as well as 13 tentatively screened OHAs, were analyzed in orange, mandarin, grapefruit and pomelo juices using our newly developed high-resolution HPLC-UV and fluorescence detection method. Quantitative OHA profiles from 158 pure juice samples were obtained to establish a purity discriminant model using an omics strategy. Reduction of OHA variables showed that three important methoxyflavones, i.e. isosinensetin, tangeretin and sinensetin provided the best discrimination ability between sweet orange and mandarin juices. There are two subtypes of pomelos, Shatianyou Group and Wendan Group, of which juices should be separately compared to grapefruit juice. Five OHAs, namely meranzin, 3,5,6,7,8,3',4'-heptamethoxyflavone, osthole, 6',7'-epoxybergamottin, and bergamottin were found to discriminate Shatianyou Group of pomelo juice from grapefruit juice; whereas three OHAs, namely bergaptol, isomeranzin, and 6',7'-dihydroxybergamottin were able to discriminate Wendan Group of pomelo juice from grapefruit juice. The established partial least squares discriminant analysis (PLS-DA) models were capable of detecting as little as 10% mandarin juice in sweet orange juice and 10% pomelo juice in grapefruit juice, allowing for fast prescreening of excess addition with good reliability (root mean square error of prediction, RMSEP < 5%).

## Introduction

Orange (*Citrus sinensis*) and grapefruit (*Citrus paradisi*) juices are among the most popular single-fruit juices worldwide. In the USA, commercial orange juice has been legally defined as juice obtained by a mechanical process from the endocarp of *Citrus sinensis*, in which mandarin (*Citrus reticulata*) or its hybrids are allowed to be added in an amount not to exceed 10% by soluble solids (1, 2) or volume (3). In Europe, no mandarin addition is allowed. A similar definition of grapefruit juice can be found in the Codex and US Code of Federal Regulations. However, in the presence of severe diseases such as citrus greening (huanglongbing, HLB) and canker, fruit supplies have been reduced, and the cost of growing oranges and grapefruit is increasing. With higher prices and less availability conflicting with high consumer demand, the juice industry is vulnerable to adulteration. Among the common adulterations, adding excess inexpensive citrus juice but not declaring this addition on the label is covert and difficult to detect. To identify such adulteration, numerous methods have been developed that employ techniques such as isotopic analyses, nuclear magnetic resonance (NMR) spectroscopy, tracer addition, UV/vis-spectrophotometry, inductively coupled plasma-atomic emission spectrometry (ICP-AES), and electrochemical detection. These studies were critically evaluated by Widmer et al. in the 1990s (4). In our previous study, we established an identification model by using principal component analysis of 32 volatile organic compounds in both sweet orange juice and mandarin juice, and the model can visually identify a blending of mandarin juice at the volume fraction of 10% or above (5). The European Fruit Juice Association (AIJN) and Sure Global Fair (SGF) have issued sets of standards for authenticity tests, which rely on multiple advanced analytical techniques.

The comprehensive sensory attributes of citrus juice such as color, sweet-sour taste, rich odor, bitterness, and astringency, constitute their decisive organoleptic and commercial value. These attributes are determined by citrus metabolites, among which secondary metabolites are the major contributors and are of particular interest. Citrus-derived secondary metabolites include flavonoids, coumarins, phenolic acids, alkaloids, limonoids, carotenoids and terpenoids. The presence of these metabolites varies among different citrus species and different fruit parts (6). Among them, there is a group of derived polyphenols that all possess a benzo oxygen-containing ring and are found primarily in the form of aglycones. These compounds are called oxygenated heterocyclic aglycones (OHAs) (7–10) and consist of methoxyflavones (MFs) which are found almost exclusively within the citrus family, and coumarins, in which furanocoumarins (FCs) are an often separately mentioned subset. OHAs also take part in a variety of bioactive roles in plants and higher mammals (11–15). In a recent study, we developed a relatively rapid analytical procedure to concentrate, separate, and accurately identify 37 major OHAs that exist at low concentration in citrus juices by using solid-phase extraction (SPE) and high-resolution HPLC with relatively affordable UV and fluorescence detections (16). We also demonstrated that the shape and details of absorbance and emission spectra can provide useful structural information for OHA identification and grouping.

Omics techniques provide new opportunities to study food quality and characteristics as a comprehensive strategy and powerful tool for the assessment of juice authenticity. Omics approaches, mainly composed of fingerprinting and profiling, combined with appropriate statistical data processing, have recently been shown to be an effective tool in citrus studies, such as the comparative study of citrus fruit peels (8, 17); the chemotaxonomy, origin and evolution of citrus species (18, 19); and fraudulence detection of citrus essential oil and juice (20–22). Fingerprinting is generally an untargeted method, whose main goal is not the identification of each detected element but providing unbiased and comprehensive data, for the purpose of detecting differences between or classifying among samples. Profiling is a targeted method in which samples are analyzed on the basis of the qualitative and quantitative distribution of selected, known markers, where the unrelated elements are not considered (23, 24). Formed in the cinnamic acid pathway, the production and accumulation of OHAs were found to be highly specific among citrus species due to the spatiotemporal expressions of related genes (18, 25). Profiling of citrus juice OHAs holds the potential for the assessment of juice authenticity.

The objective of this study was to develop OHA based discriminant models to evaluate the authenticity of single-strength (non-concentrated) orange and grapefruit juices using the strategy of targeted metabolomics and chemometrics. The first step was to identify and screen juice OHAs as many as possible and compose a target library. The second step was to quantitatively profile OHAs in sweet orange and grapefruit juices as well as those of the most likely adulterant citrus juices. The third step was to use these big data of pure juices to establish recognition models to discriminate between legally allowed addition and excess addition to sweet orange and grapefruit juices and evaluate the capability of the models as a tool for detection of such food fraud.

## Materials and Methods

### Standards and Solvents

Thirty-nine HPLC-grade standard compounds of OHAs were purchased from TRC (Canada), ChromaDex (USA), Yuanye Biotech (Shanghai China), PurifyTech (Chengdu, China) and Sigma (St Louis, USA). Supplementary Figures 1–3 in the Supplementary Materials shows their structural information. Stock solutions of the individual standards were prepared at approximately 1 mg/mL in methanol or dimethyl formamide. Alkylarylketones, C8–C14, were procured from Sigma-Aldrich (St Louis, USA), and they were utilized to prepare a mixture at a concentration of 10 mg/L each. All solvents were of HPLC-grade. Ultrapure water was freshly produced using a Milli-Q A10 system (Molsheim, France).

### Sampling, Juicing and Thermal Pasteurization

Fresh mature fruits obtained from 68 orange, 43 mandarin, 17 grapefruit, and 30 pomelo samples of 47 major commercial cultivars, were harvested from well-managed orchards in different regions. Samples from the same region were typically collected over 2–3 years or harvest seasons. Supplementary Table 1 details information on the fruit samples. Fruits were hand extracted to release the juice. The citrus juice was fine-filtrated by passing through an 80-mesh screen and then pasteurized for 60 s using a 100°C water bath.

### Fractionation and Extraction

Juice fractionation and extraction procedures followed our previous work (16). Briefly, pasteurized juice was centrifuged to separate clear serum from suspended cloudy solids. The solid deposit was thoroughly extracted using acetonitrile. The aqueous serum was solid-phase extracted by passing through a Waters C-8 cartridge, cleaned with a 73:27 (v/v) mixture of aqueous phosphoric acid /acetonitrile (pH 5.5), and retained OHAs were eluted using ethyl acetate. The acetonitrile extract and ethyl acetate extract were combined and dried under nitrogen and redissolved in pure methanol to form a 9-fold concentrated sample.

### Analytical Conditions and Detector Settings

A Poroshell 120 EC-C8 column (i.d. 4.6 × 150 mm, 2.7 μm) attached to an EC-C18 guard column (i.d. 4.6 × 5 mm, 2.7 μm) was used with an Agilent 1,260 chromatography system equipped with a diode array detector (DAD) and a fluorescence detector (FLD) in series. The column temperature was maintained at 30°C. The mobile phases consisted of 0.05% phosphoric acid/water (A), methanol (B), acetonitrile (C), and a mixture of water/acetonitrile/tetrahydrofuran (volume ratio 55/20/25, D). The flow rate of mobile phase was 1 mL/min, and the separation gradient consisted of the following linear steps: 0–7 min, 33% B, 4% C and 0% D; 7–9 min, 33–32% B, 4–5% C and 0–6% D; 9–17 min, 32% B, 5% C and 6% D; 17–19 min, 32–22% B, 5% C and 6–26% D; 19–29 min, 22–8% B, 5–19% C and 26% D; 29–32 min, 8–0% B, 19–34% C and 26% D; 32–35 min, 0% B, 34–41% C and 26% D; 35–40 min, 0% B, 41–73% C and 26–0% D; 40–45 min, 0% B, 73% C and 0% D; 45–50 min, 0% B, 73–100% C and 0% D; and 50–75 min, re-establishing initial conditions. During the whole gradient solvent A was always used to make up the composition to 100%. The DAD was set to scan 210–400 nm and monitor UV responses at 330, 270 and 250 nm. The FLD had an excitation wavelength of 340 nm, and emission spectra were record between 340 and 560 nm. Three fluorescence emission signals were monitored at 400, 450, and 500 nm.

### Identification and Screening of OHAs

The established and validated identification method was applied to this study (16). Absorbance and emission spectra of each standard compound were determined and compiled in a in-house library. First, each entire spectrum was used to confirm peak purity and identity by checking the conforming degree of the sample peak compared to standards. Second, fluorescence peak height ratios at different monitored wavelengths were calculated as an additional identification tool. Third, the retention index (RI) values were determined using the C8–C14 series of alkylarylketones and calculated using the programmed temperature RI equation (26). RIs of sample peaks were compared with the standard to further ensure the accuracy of identification. In addition, UV and fluorescence entire spectra as well as the fluorescence numeric ratios of unknown peaks were compared with the established library for screening of OHA candidates.

### Quantitation and Validation

Quantitation of the samples was carried out using a calibration equation in the linear range. Calibration curves were constructed with seven concentrations, in which the lowest concentration was that showing a signal-to-noise (S/N) ratio between 3 and 10, whereas the highest concentration was that maintaining a linear correlation coefficient (R^2^) ≥ 0.999. The correction factor, determined using 5 mg/L psoralen as the internal standard (IS), was used against any biased results.

Linearity, limit of detection (LOD), limit of quantitation (LOQ), intraday and interday repeatability, and recovery were determined for validation of the sample preparation and quantitation methods. To simplify the evaluation of repeatability and recovery, analyte surrogates (ASs), such as coumarin (for coumarins), xanthotoxin (for FCs) and gardenin A (for MFs), were added to juice samples at a final concentration of 5 mg/L. The 39 mixed OHA standards and three surrogates were repeatedly injected within and across different days. Intra- and interday repeatability of the surrogates were determined, and the results were used to represent precisions of the analogous OHAs. Ten juice samples were randomly selected to study the surrogate recovery.

### Statistical Analysis

All analyses were performed in triplicate, and the results were processed using SPSS 22 (IBM, USA). The data matrix, made up of OHA variables in columns and juice samples in rows, was analyzed by ANOVA and Duncan's test (*p* < 0.05). Multivariate analysis was further performed on The Unscrambler X10.4 (Camo, Norway) using principal component analysis (PCA) and partial least squares discriminant analysis (PLS-DA).

## Results and Discussion

### Qualitative Analysis and Tentative Identification of Juice OHAs

We demonstrated in our recent work that by matching an innovative combination of full UV and fluorescence spectra, multiwavelength fluorescence emission peak ratios, and alkyl aryl ketone LC-RI values with those of OHA standards, minor peaks of juice samples with low instrumental responses can be identified (16). A more comprehensive investigation of juice OHAs is favorable when applying the strategy of targeted metabolomics and the measurement of defined groups of chemically characterized or annotated components. Unidentified OHAs other than the 39 known compounds are constituent part of the whole profile and could contribute to the assessment model. Therefore, we further sought unknown OHAs in juice samples. This analysis is commonly performed by using the expensive high-resolution mass spectrometry such as orbitrap or q-ToF screening, as they provide accurate mass information of the parent and fragment ions, revealing a few structural characteristics. In the current study, we were able to tentatively confirm their existence by using the more affordable DAD and FLD and categorize these compounds into structural subtypes by comparing the UV and fluorescence full spectra with those of the known OHAs.

#### Spectral Characteristics Corresponding to Fine Structures

**Figure 1:**
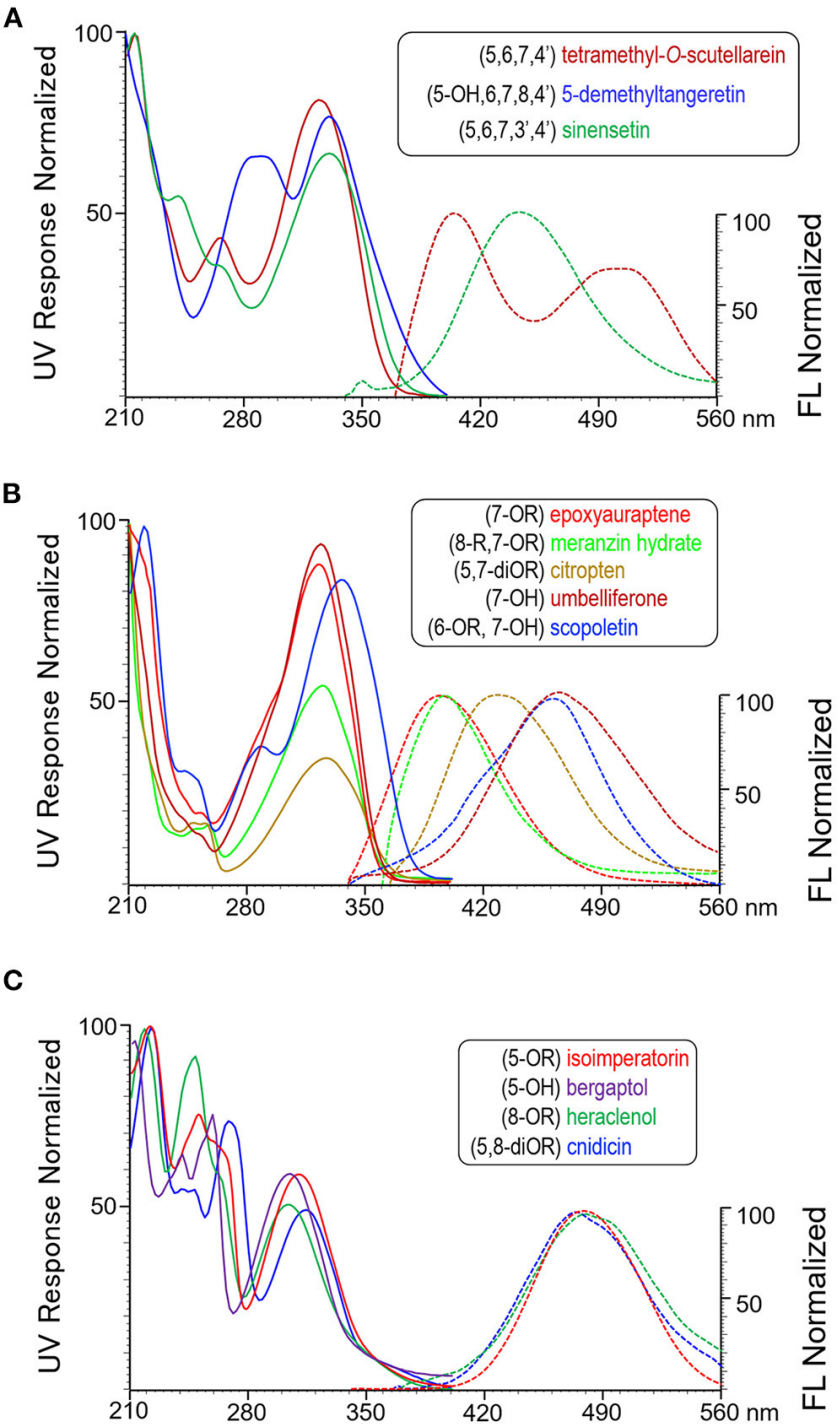
Representative UV absorbance and fluorescence emission spectra of the subgroups of three OHA families, **(A)** methoxyflavones, **(B)** coumarins, and **(C)** furanocoumarins. UV spectra are shown in solid lines and ranged between 210 nm and 400 nm. Fluorescence spectra are shown in dash lines and ranged between 340 nm and 560 nm. All signals are normalized.

Methoxyflavones can be subcategorized as 4'-methoxylated MFs and 3',4'-dimethoxylated MFs. As shown in Figure 1A, each of the representatives (tetramethyl-*O*-scutellarein and sinensetin) shows a single and double shoulder peak around 240–280 nm of the UV spectra. Additional 5-hydroxylation shifts UV spectrum toward longer wavelength and eliminates fluorescing as shown by 5-demethyltangeretin.

Coumarins are structurally categorized according to the number and position of the substituent groups. Nuances of UV and fluorescence spectra among subcategories are shown in Figure 1B. 7-OR (epoxyauraptene) and 7-OR,8-R disubstituted (meranzin hydrate) coumarins both have UV and fluorescence maxima at approximate 323 nm and 395 nm; however, the latter one also shows a small shoulder in the UV spectrum near 256 nm. 5, 7-DiOR substitution as represented by citropten shifts both UV and fluorescence maxima toward longer wavelengths. Compared with the 7-OR coumarin, 7-hydroxylation as shown by umbelliferone greatly red-shifts emission maximum to 461 nm but has little effect on UV maximum wavelength. Additional -OR substitution such as for scopoletin (6-methoxy, 7-OH) shifts UV maximum toward longer wavelength (323 nm 336 nm) but does not significantly change the fluorescence maxima.

Similar to coumarins, spectral shape and characteristics of furanocoumarins represent their substituent number (1 or 2) and position (5- and/or 8-) on the parent ring. As shown in Figure 1C, all the UV spectra possess a large shoulder peak and a comparatively smaller main peak. The shoulder maximum wavelength of mono-substituted (5- or 8-OR) FCs is approximate 250 nm, and that of 5,8-diOR substituted FC is 270 nm. The main peak of 5-OR FC is approximate 310 nm, whereas those of 8-OR and 5,8-diOR FCs are shorter and longer than 310 nm, respectively. Most FCs show FL emission maxima close to 480 nm. Particularly, 5-hydroxylation quenches fluorescing and alters the profile of UV spectrum subtly, as shown by bergaptol.

#### Screening of Unknown OHAs

**Figure 2:**
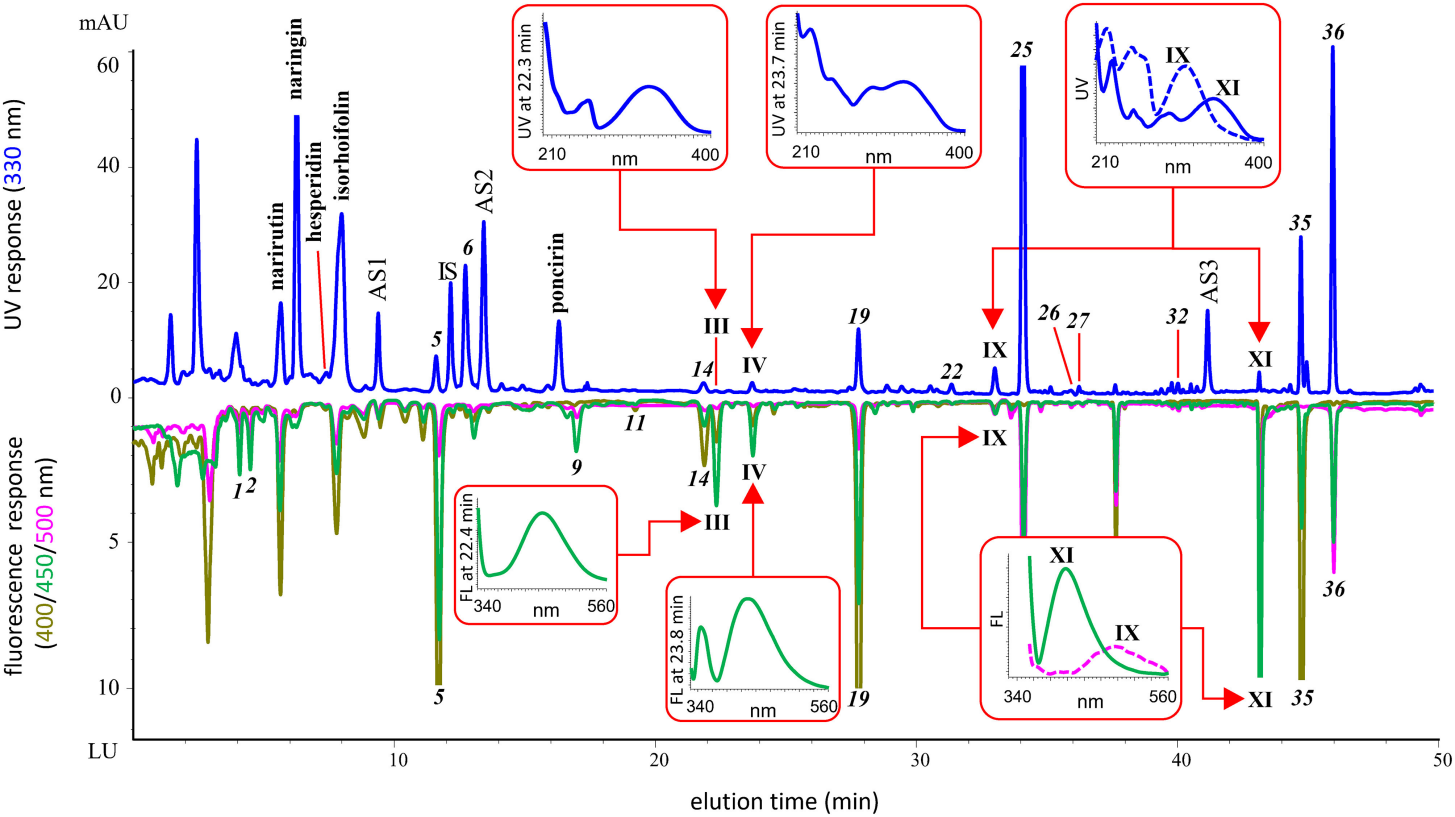
Chromatograms of identified OHAs, tentatively identified OHA candidates and other major components in Thompson grapefruit juice. Showing the UV and fluorescence spectra of the tentatively identified OHAs.

By using the spectra-structure empirical rules and carefully checking the featured numeric database of multiwavelength fluorescence emission peak ratios, additional 13 OHA candidates were screened out from all the juice samples. Their UV and fluorescence spectral maxima and monitored fluorescence peak height ratios are shown in Table 1. The RI values were also calculated to establish the temporal identity of the compounds, and all the values are different from the 39 known OHAs. Generally, the 13 candidates were tentatively identified as 8 coumarins and 5 FCs. Coumarins were found in all the four juices while FCs were typically detected in grapefruit and pomelo juices. Figure 2 shows an example of the identified major components and tentatively identified OHAs in Thompson grapefruit juice. We detected four OHA candidates, i.e. coumarins III, IV and XI, and an FC IX. Their UV and fluorescence full spectra are shown in the figure. Compound III shows a similar UV spectrum to that of citropten *(9)*, a 5,7-diOR coumarin, but its maximal emission wavelength (463 nm) is longer than that of citropten (437 nm), suggesting that the 7-position is possibly hydroxylated and the molecular conjugation system is enhanced. Similarly, compound XI, of which UV spectrum is almost identical to scopoletin *(1)* but emission maximum 40 nm shorter, was tentatively identified as a 6,7-diOR coumarin. By comparing XI's UV spectrum with that of 6,7-dimethoxycoumarin as reported by Christie and Lui (27), the deduced substitution pattern is further confirmed. Finally, the tentative substitutive structure of all the 13 OHA candidates are shown in Table 1 with supporting references.

**Table 1:** Retention index, UV and fluorescence spectral maxima, and fluorescence peak height ratios of the 13 tentatively identified OHAs.

**No**.	**Rt (min)**	**RI**	**UV absorbance max (nm)**	**Emission max** **(nm)**	**Highest / 2^**nd**^ highest monitored FL peak (nm)**	**FL peak height ratio**	**Deduced substitution pattern**	**Representative** **samples**
I	5.5	NA	230, 295, **347**	462	450 / 500	2.3	6-OR, 7-OH C^*^	mandarin
II	15.0	877	262, **331**	467	450 / 500	1.4	5-OR, 7-OH C (28)	all juices
III	22.3	958	262, **330**	463	450 / 500	1.6	5-OR, 7-OH C (28)	grapefruit
IV	23.7	973	224, 248, 295, **330**	431	450 / 400	1.6	uncategorized C	grapefruit
V	26.8	1006	259, **328**	457	450 / 500	2.1	5-OR, 7-OH C (29)	orange
VI	28.0	1018	252, 258, **326**	454	450 / 500	2.2	5,7-diOR C^*^	orange
VII	29.0	1028	230, 295, **344**	422	450 / 400	1.1	6,7-diOR C (27)	pomelo
VIII	31.1	1049	223, **251**, 312	476^†^	450 / 400	1.8	5-OR FC^*^	grapefruit
IX	33.0	1067	222, **251**, 312	488	500 / 450	2.2	5-OR FC^*^	grapefruit
X	40.7	1260	222, **251**, 309	485^†^	500 / 450	1.6	5-OR FC^*^	grapefruit
XI	43.0	1313	227, 294, **343**	422	450 / 400	1.1	6,7-diOR C (27)	grapefruit
XII	43.3	1318	221, **250**, 308	477^†^	450 / 500	1.4	5-OR FC^*^	grapefruit
XIII	43.9	1331	223, 242, 249, **268**, 311	496	500 / 450	4.7	5,8-diOR FC^*^	pomelo

*Bold digits show the wavelength of the maximal response of UV absorbance spectra. C, coumarin; FC, furanocoumarin; MF, methoxyflavone; -OR, alkoxy group*.

* *Substitution pattern refers to the known OHAs*.

† *Low emission responses. NA, not available to be calculated because this compound eluted before the first alkylarylketone standard, propiophenone (C8)*.

### Quantitation of OHAs and Method Validation

Basically, MFs and coumarins were quantified using UV detection at 330 nm, 5- or 8- monosubstituted FCs at 250 nm, and 5,8-disubstituted FCs at 270 nm. Three wavelengths, 400, 450 and 500 nm, were used for fluorescence quantitation as a compromise of convenience and sensitivity. With a few exceptions, MFs were quantified at 450 nm, coumarins at 400 and 450 nm, and FCs at 500 nm. The screened OHA candidates were quantified similarly, and the results are expressed using their response peak area.

The linear ranges, equations and correlation coefficients (R^2^) for both UV and fluorescence responses were determined under the established conditions. As shown in Table 2, each equation has good linearity (R^2^ > 0.9990) within the stated concentration range. LODs and LOQs were determined at S/N ratios of 3 and 10, respectively. All LOQs ranged between 0.04 and 1.05 mg/L when using UV detection, and most of LOQs were below 1.0 mg/L. Emission LOQs were highly variable, ranging from 0.0004 to 35 mg/L. Fluorescence detection is a powerful tool for the trace determination of coumarins, of which LOQs are 3–100 times more sensitive than UV detection. However, it was completely incapable of detecting certain few OHAs such as bergaptol and isosinensetin, as they produce no useable fluorescence emission signal.

**Table 2:** Calibration and quantitative parameters of the 39 OHAs.

**No**.	**Compound**	**UV quantitation**						**Fluorescence quantitation**					
		**Range (mg/L)**	**Slope of equation**	**R^**2**^**	**LOD (mg/L)**	**LOQ** **(mg/L)**	**CF**	**Range (mg/L)**	**Slope of equation**	**R^**2**^**	**LOD (mg/L)**	**LOQ** **(mg/L)**	**CF**
1	scopoletin	0.05–500	5.2828	0.9992	0.01	0.04	10	0.0005–25	37.891	0.9998	0.0002	0.0007	12
2	umbelliferone	0.05–500	4.1033	0.9998	0.01	0.04	8.1	0.001–5.0	84.201	1.0000	0.0001	0.0004	27
3	herniarin	0.5–600	3.9501	0.9996	0.31	1.05	7.8	0.1–300	5.0011	0.9995	0.02	0.08	0.56
4	heraclenol	0.2–500	3.4774	1.0000	0.15	0.51	18	30–1200	0.0272	1.0000	10.5	35	0.0036
5	meranzin hydrate	0.5–500	2.7524	0.9999	0.16	0.55	5.4	0.02–100	9.5500	1.0000	0.0074	0.025	1.1
6	bergaptol	0.1–500	4.4802	0.9997	0.02	0.08	23	–	–	–		–	–
7	oxypeucedanin hydrate	0.5–500	3.1312	1.0000	0.16	0.52	6.1	2.0–2000	0.0650	0.9999	0.83	2.77	0.0086
8	byakangelicin	0.5–150	2.7260	0.9999	0.02	0.06	3.8	5.0–500	0.2071	0.9998	0.64	2.13	0.028
9	citropten	0.1–500	6.2133	1.0000	0.02	0.06	12	0.2–500	54.720	0.9996	0.0004	0.0016	17
10	bergapten	0.3–500	3.8002	0.9994	0.03	0.10	20	1.0–500	0.1364	0.9999	0.13	0.43	0.018
11	auraptenol	0.5–300	3.4638	0.9999	0.17	0.56	6.8	0.03–100	8.7202	1.0000	0.01	0.03	0.97
12	heraclenin	0.5–1200	3.1276	1.0000	0.22	0.73	16	5.0–1200	0.0203	1.0000	2.60	8.67	0.0027
13	meranzin	0.2–500	4.1710	0.9997	0.04	0.12	8.2	0.2–500	5.5027	0.9996	0.0012	0.0050	0.61
14	isomeranzin	0.05–500	4.3205	0.9996	0.04	0.14	8.5	0.01–50	5.4720	0.9986	0.0023	0.0075	6.1
15	isosinensetin	2.0–1000	2.7304	0.9995	0.11	0.36	5.4	NA	NA	NA	NA	NA	NA
16	byakangelicol	1.0–4000	3.0139	1.0000	0.20	0.67	4.2	NA	NA	NA	NA	NA	NA
17	sinensetin	5.0–500	3.0462	1.0000	0.27	0.92	6.0	5.0–500	0.8602	0.9995	0.13	0.40	0.27
18	oxypeucedanin	1.0–500	4.6982	0.9995	0.04	0.14	25	5.0–500	0.2071	0.9998	0.13	0.43	0.066
19	marmin	1.0–500	2.8562	0.9998	0.02	0.07	4.9	0.2–500	3.9527	0.9998	0.01	0.03	0.30
20	hexamethyl-O-quercetagetin	1.0–800	1.7258	0.9997	0.08	0.27	3.4	5.0–800	0.4830	0.9992	0.08	0.25	0.15
21	tetramethyl-O-isoscutellarein	1.0–1000	2.8129	0.9999	0.04	0.12	5.5	NA	NA	NA	NA	NA	NA
22	nobiletin	2.0–500	5.0870	0.9998	0.09	0.30	10	5.0–500	0.2072	0.9998	0.15	0.51	0.066
23	tetramethyl-O- scutellarein	1.0–5000	6.0807	0.9998	0.06	0.19	12	5.0–5000	0.1875	0.9996	0.25	0.85	0.021
24	3,5,6,7,8,3',4'-heptamethoxyflavone	1.0–500	6.1161	0.9998	0.03	0.11	12	10–500	0.0693	0.9998	0.41	1.44	0.022
25	6',7'-dihydroxy-bergamottin	0.1–1000	2.1900	0.9999	0.01	0.04	11	1.0–600	0.0910	0.9990	0.04	0.15	0.012
26	tangeretin	0.5–500	6.7485	0.9994	0.02	0.06	13	–	–	–	–	–	–
27	5-demethylnobiletin	0.5–1000	3.1097	0.9998	0.04	0.13	6.1	–	–	–	–	–	–
28	imperatorin	0.2–200	2.5970	0.9998	0.02	0.05	14	15–1500	0.0085	0.999	4.77	15.9	0.0011
29	phellopterin	0.2–200	3.3218	0.9996	0.01	0.04	4.6	NA	NA	NA	NA	NA	NA
30	5-demethyl- tangeretin	0.5–500	2.0667	1.0000	0.11	0.38	4.1	–	–	–	–	–	–
31	osthole	0.5–500	4.4870	0.9999	0.02	0.08	8.8	1.0–500	1.9800	0.9998	0.05	0.15	0.22
32	isoimperatorin	0.2–500	4.0122	0.9998	0.01	0.03	21	1.5–3000	0.0911	1.0000	0.27	0.89	0.012
33	6',7'-epoxy- bergamottin	1.0–500	2.8260	0.9998	0.04	0.13	15	2.0–500	0.1563	1.0000	0.11	0.38	0.050
34	8-geranyloxy- psoralen	0.2–200	1.9664	0.9999	0.01	0.04	10	20–500	0.0028	0.9994	10.2	24.0	0.00040
35	auraptene	1.0–500	2.3146	0.9999	0.03	0.11	4.5	0.2–500	2.6926	0.9998	0.01	0.03	0.30
36	bergamottin	0.5–500	4.3068	0.9997	0.02	0.06	22	0.5–500	0.5795	0.9999	0.02	0.06	0.18
37	5-geranoxy−7-methoxycoumarin	0.5–500	2.7850	1.0000	0.12	0.40	5.5	0.02–50	20.998	0.9999	0.005	0.020	6.7
38	6',7'-epxoy- auraptene	3.2–1600	4.7086	0.9999	0.06	0.25	12	0.6–1600	6.2310	0.9998	0.02	0.06	0.70
39	cnidicin	0.2–300	3.3762	0.9999	0.02	0.07	5.4	NA	NA	NA	NA	NA	NA

*NA, not available for quantitation because of the low emission response; - fluorescence emission not detected*.

Coumarin, xanthotoxin and gardenin A, numbered ASs 1–3, were used as surrogates for the precision and recovery test. These ASs were carefully chosen, as they are not naturally contained in citrus juice samples, cover the wide polarity range of OHAs, and are retained in the major fraction after SPE pretreatment. The current analytical method showed very good inter- and intraday repeatability (absolute relative error ≤ 2.1%) and recovery rate (within 98 and 102%) for each surrogate, as presented in Supplementary Table 2.

### Quantitative Profile of OHAs Among Four Citrus Juices

The major juicing cultivars of sweet orange and grapefruit and the possible additive cultivars from mandarin and pomelo, 158 samples of 47 cultivars in total, were extracted, and the juices were heated at 100°C for 60 s to mimic the industrial high-temperature short-time (HTST) pasteurization conditions. Each heated juice was quantified for the 39 known OHAs and also subjected to determination of the 13 tentatively screened OHA distributions and peak areas.

**Figure 3:**
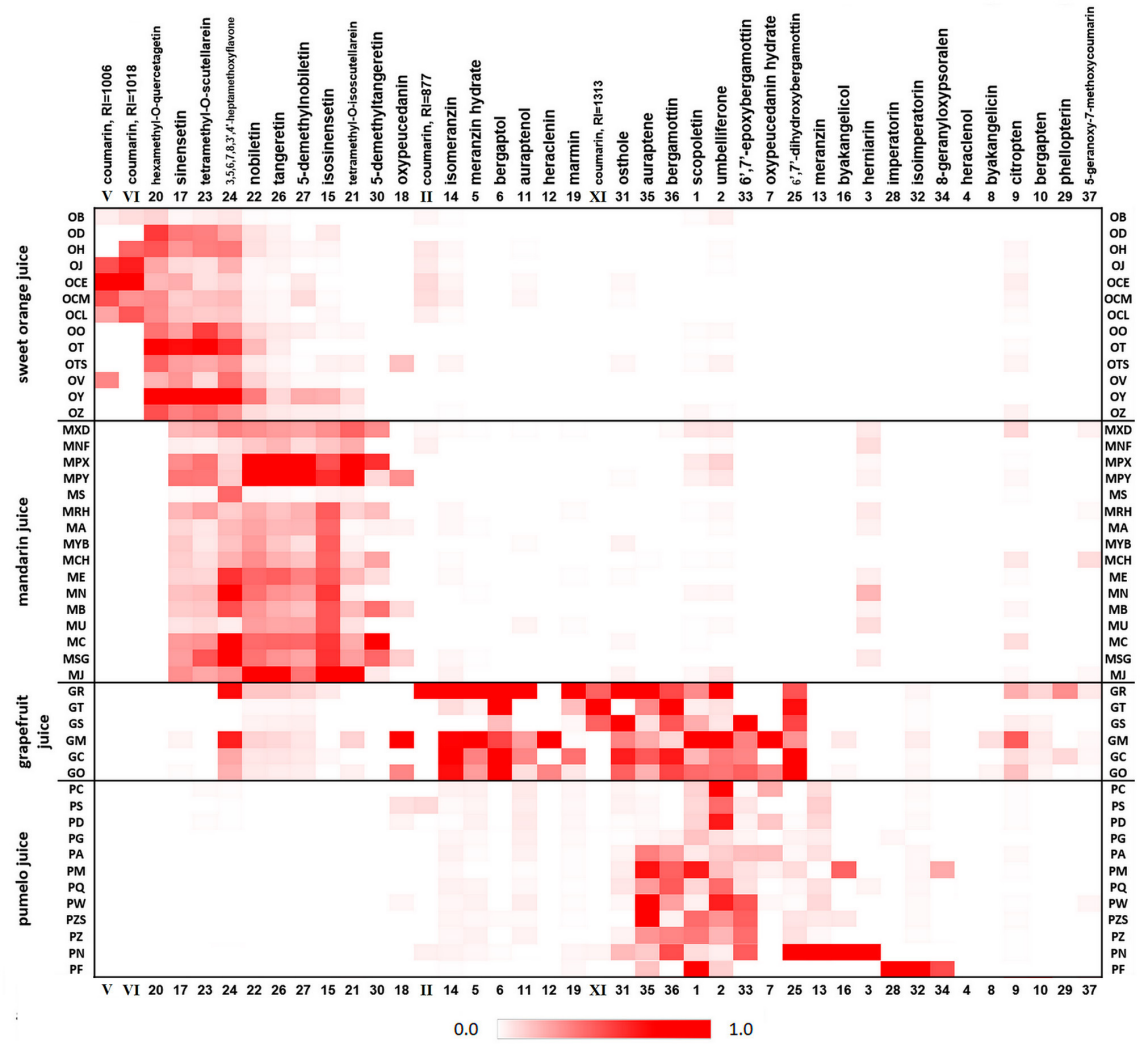
Heat map displaying OHAs distribution profile and relative weight in the pasteurized juices of 47 citrus cultivars. Concentration of each compound was normalized across all samples, the highest amount set as 1.0, to better show the interspecific difference. Compounds II, V, VI, and XI, the tentatively identified coumarins, were calculated using their fluorescence emission responses. Abbreviated name for juice samples correspond to Supplementary Table 1.

The total amount of juice OHAs varied among species. Sweet orange juices normally contained these compounds at < 5 mg/L; mandarin juice averaged 5–10 mg/L; and pomelo and grapefruit juices ranged among 5–100 mg/L. Due to the large concentration variance over different varieties and even samples of the same species, it was difficult to conclude the OHA distribution characteristics by direct comparison. Therefore, the quantitative results were normalized for each compound across all the cultivars, expressed as relative weight, and shown in the form of a heat map (Figure 3) where crimson represents the highest weight, 1.0, and the color intensity decreases as the relative weight (normalized concentration) decreases. 6,7-Epoxyauraptene *38* and cnidicin *39* were excluded because they were found at trace concentrations in very few juices; four of the tentatively identified coumarins (II, V, VI and XI) were included, as they were found in many samples. All compounds were rearranged to better show their clustering in juice varieties.

Sweet orange and mandarin juice OHA profiles were characterized mainly by large amounts of MFs distributed in the upper-left region of the heatmap. However, the hot spots varied between juice type: sweet orange juices, compounds *20, 17, 23* and *24* and mandarin juices, compounds *24, 22, 26, 27, 15, 21* and *30*. Among them, heptamethoxyflavone *24* was detected at high concentrations in both orange and mandarin juices. We also screened two candidates (V and VI) of 5,7-disubstituted coumarins exclusively contained in orange juices. A 6,7-disubstituted coumarin, compound I, was found to be almost universally distributed in all varieties of four juices.

Highly clustered regions of grapefruit and pomelo juices were located in the lower-middle of the heatmap. These juices had similar constituents of coumarins and FCs, covering almost all compounds from *18* to *37*. Grapefruit juice contained more OHAs *18*–*31* than pomelo juice, including the tentatively identified 6,7-diOR coumarin XI. These juices had similarly high levels of compounds *35*–*33* (all compound numbers follow the order in which they appear in the heatmap). In addition, seven MFs were found at medium-high levels in grapefruit juices, whereas only five of them, compounds *23*–*27*, were detected at very low levels in pomelo cultivars Changshoushatianyou (PC), Shantianyou (PS) and Dayongjuhuaxin (PD), all related to the Shatianyou variety, a well-known group of cultivars with relatively low acidity but high sugar contents.

### Identification of Orange and Grapefruit Juices Adulterated by Mandarin and Pomelo Juices

Regarding the adulteration of citrus juices worldwide, the most common practice is the excessive incorporation of mandarin juice into sweet orange juice. This adulteration could be highly profitable in China, where mandarin fruits are produced in large quantities and are commonly sold at an inexpensive price. Although the mandarin-specific marker, 5-demethyltangeretin (16), can be used to detect if mandarin juice was incorporated, the large variation in its content among cultivars makes it impossible to determine the blended proportion. Therefore, to quantitatively determine blending fraudulence, an exhaustive data structural analysis of the characteristic OHAs of orange and mandarin juices should be applied. The same strategy was used when identifying whether relatively expensive grapefruit juice was adulterated with the comparatively inexpensive pomelo juice.

#### Prescreening of OHAs to Discriminate Juice Variety

To understand how these compounds could differentiate citrus juices corresponding to species, PCA was first applied to study the whole quantitative data structure. Basically, all data were mean centered and autoscaled to form a 158 × 41 matrix, in which 37 OHAs and 4 tentatively identified coumarins were variables. The results showed that four juice species were separated with 21 known OHAs and a coumarin candidate XI. Specifically, orange and mandarin juices were separated by seven MFs, compounds *15, 17, 21, 22, 23, 26* and *27*. Grapefruit and pomelo juices were separated by 5-substituted FCs such as compounds 6, *25*, and *36* and disubstituted coumarins such as compounds *5, 11, 14, 19, 31, 35* and XI. These results demonstrated the feasibility of separating each juice variety using the quantitative profiles of the 22 OHAs. Seven and 10 OHAs were prescreened for further study of their discriminant ability for sweet orange-mandarin juice blending and grapefruit-pomelo juice blending, respectively.

#### Detection of Sweet Orange Juice Incorporated With Mandarin Juice

##### Variable Reduction

**Figure 4:**
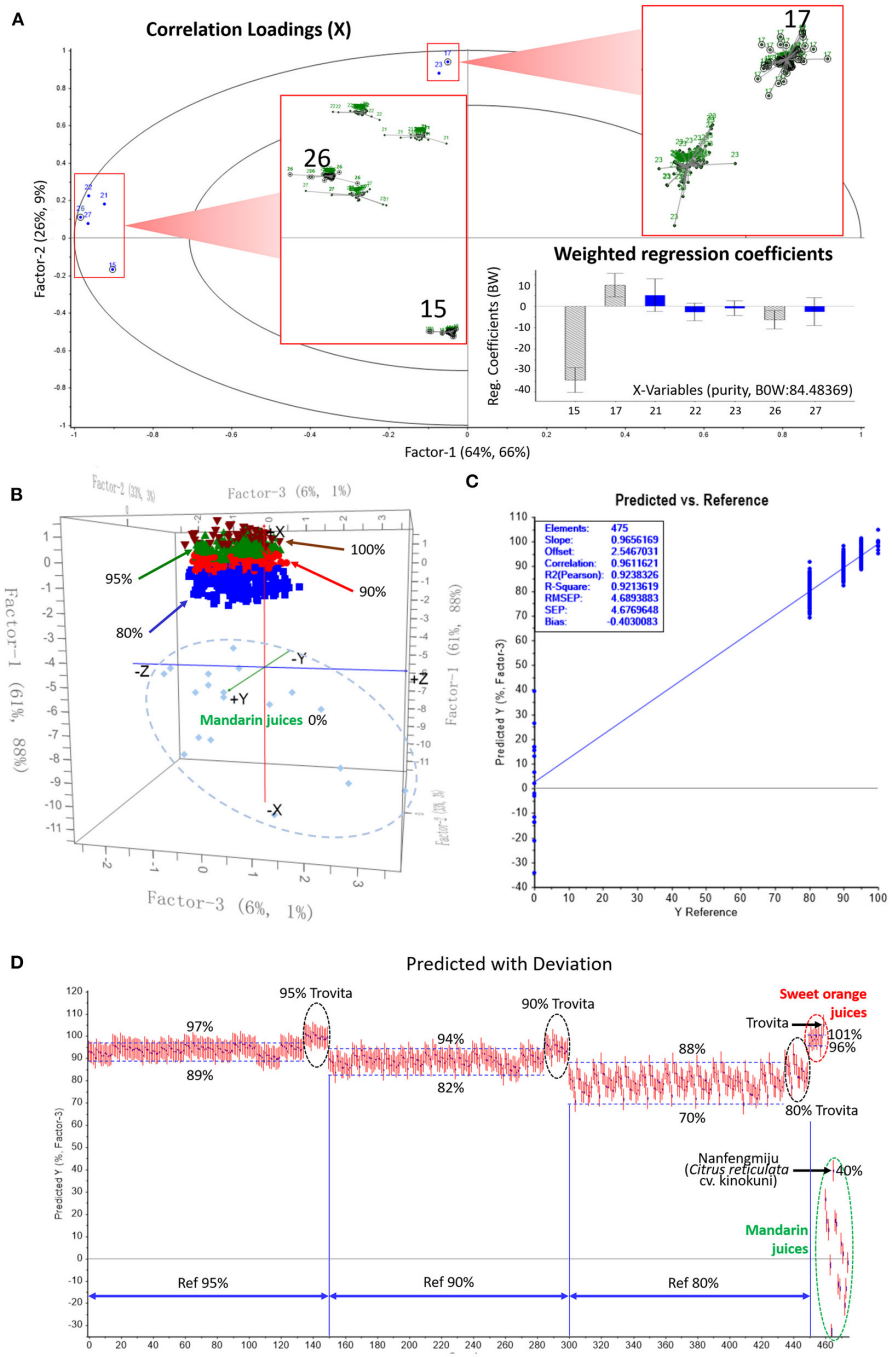
Partial least squares discriminant analysis (PLS-DA) of sweet orange juice incorporated with different proportion of mandarin juice. **(A)** Methoxyflavone variables reduction according to the uncertainty and regression coefficients; **(B)** Score plot of training set showing clustering of different mixtures of sweet orange and mandarin juice; **(C)** Prediction ability of the PLS regression model evaluated by external validation; **(D)** Prediction results of orange juice purity in mixed juices.

Concentrations of the seven characteristic MFs in 65 sweet orange and 40 mandarin juice samples were used for further reduction of variables. To be noted, Satsuma mandarin (*Citrus unshiu* Marc.), which is well-known to produce strong sulfuric off-flavors after heating and storage (30), is not often used for addition into orange juice. Therefore, the data of Satsuma juices were not included. This 105 × 7 matrix was analyzed with the PLS regression method, applying the full cross-validation and the Martens' uncertainty test. The results illustrating the importance of variables are shown in Figure 4A. Although all seven MFs had high correlation loadings (R^2^ > 0.9) in the PLS model, their stability, which is expressed as the sum of the squares of the differences in all submodels (enlarged displayed), was different. The more converged each submodel was, the less uncertain the variable. The difference was also shown in terms of the weighted regression coefficients at the lower right corner. Variables shown as blue columns have uncertainty limits crossing the zero line of Y ordinate, indicating that they are not significant at the 5% level with a 95% confidence interval preset. Gray striped columns are either above or below the zero line, showing that they clearly have a significant effect on Y values. Therefore, isosinensetin *(15)* and tangeretin *(26)* were significant markers of mandarin juice, and sinensetin *(17)* was a significant marker of orange juice.

##### Construction of Recognition Model

A training set was constructed using the concentrations of the three important variables in pure sweet orange and mandarin juices to virtually compose those of orange juices mixed with mandarin juice at percentages of 5, 10, and 20%. Their purities, 95, 90, and 80%, were set as responses Y. Exhaustive combinations were prepared with all harvest-year averages of each available cultivar from different producing areas among the two juice groups. The data set consisted of 912 blended juice samples, 304 for each purity level. Pure juice samples were also put into the training set. Full cross-validation was applied to develop the PLS recognition model.

As shown in Figure 4B, the suggested optimal number of latent variables was three, in which Factors 1 and 2 summarize most of the variance in the whole data set. In this three-dimensional score plot, the blend clusters were stacked layer-by-layer as purity increased. Each cluster was generally well separated from its adjacent clusters; however, there was no explicit boundary, as a few samples might protrude into the neighboring cluster. This was mostly due to the dispersive distribution of mandarin juice samples, as shown in the 0% ellipse. The abundant germplasm diversity of mandarin resulted in large variance in the production of secondary metabolites. It can be further deduced that, as the blended mandarin juice proportion increased, juice mixtures showed higher dispersion so that the discrimination between such nearing groups lost efficacy. Nevertheless, the samples of 90% purity were totally separated from the 100% group, demonstrating that this model is operational to discriminate a 10% addition of mandarin juice, the minimum adulteration.

##### Prediction and Validation Using the Model

A new batch of fruits including 10 cultivars of sweet orange and 15 cultivars of mandarin were obtained in 2019 from local producers and markets. These fruits were mostly the same cultivars used to construct the training set but also included a few new cultivars to assess the model's prediction ability when processing unknown samples (see Supplementary Table 1 for information on testing samples). Juice was pasteurized, prepared and analyzed, and the isosinensetin, sinensetin and tangeretin data were collected. An external test set was constituted by an exhaustive combination of the newly analyzed juices, just as for the training set. The total number of samples in the test set was 475, including 25 pure juices. The purity of each sample predicted by using the discriminant model was compared to the reference value, the known orange juice percentage used for virtual blending.

The prediction ability of the established PLS regression model was evaluated by root mean square error of prediction (RMSEP), which is an indicator of the average error in the analysis for each component and how well the model fits the data. Figure 4C shows the RMSEP in the external validation, 4.69, which is close to 3.88, the root mean square error of cross-validation (RMSECV) of the training set. Pearson's R^2^ value of the regression model was 0.924 in the test set vs. 0.960 in the training set. The close concordance of these key parameters between prediction and cross-validation indicated that the regression model was robust.

Figure 4D shows the predicted purity (PP) vs. the reference value of the external samples. At the top right corner, samples of pure sweet orange juice were predicted to be 96%−101% pure, which matched well with their true values. The only exception was Trovita orange juice, which was predicted to be 105% pure. Trovita is considered a mutant from a seedling of Washington navel orange; this subtle genetic variance from normal sweet orange might result in a different MF profile, and its sample might be an outlier beyond the group. Correspondingly, 15 mixed samples blended with Trovita juice at three proportions (black circles) were recognized as outliers.

Compared to sweet oranges, mandarins are progenies of more ancient wild species and have accumulated more diversity. Large variation from −34% (MPX) to 40% (MN) of the PP, in terms of orange juice, of absolute mandarin juices was observed, as marked in the green circle in Figure 4D. Nanfengmiju (MN), also known as *kinokuni*, a long-cultivated local breed, is among the largest consumed mandarins in China. This cultivar is better for juicing than Satsuma because it has a better aroma and far less sulfuric off-flavor after heating, which enables its juice to be a potential adulterant for application in sweet orange juice. The 40% similarity judged by the MF model made its overblending more difficult to identify. For example, due to its participation, the predicted upper value for the 80% orange juice group was elevated from 85 to 88%, which almost reached the 90% reference line. Likewise, to explicitly identify a mandarin juice content lower than 10%, the legal additive maximum, the predicted value should be higher than 94%, which might result in a false determination of adulteration.

In food chemistry, the main problem for discriminant analysis is class overlap (31). It is especially difficult to deal with quasi-continuous classes, such as groups made of different percentages of adulterants in this study. The overlapped classes can be differentiated linearly, but a 100% discrimination may not be expected (32). Therefore, based on the predicted and validation results in the case of mandarin juice as the adulterant, we suggest the following criteria using the current developed MF model for a preliminary assessment of sweet orange juice authenticity:

1) If the PP of an orange juice product is no <94%, its labeling as “sweet orange juice” which indicates no more than 10% mandarin juice blended, can be generally regarded as authentic;
2) If the determined PP is <82%, the labeled orange juice should be evaluated as fraudulently mislabeled;
3) If the PP value is between 82 and 94%, additional analytical methods are required to further verify the juice authenticity.

A few studies used chemometric methods to determine juices authenticity. Wang and Jablonski detected lemon juice adulteration by using targeted and non-targeted LC-MS analyses and a PCA model (33). Stander et al. took a survey on South African fruit juices' adulteration using a HILIC-MS and PCA method (34). Our previous study used GC-MS and PCA methods to analyze blending of mandarin juice into orange juice (5). However, PCA can only exhibit clustering and separation visually, and it is incapable of detecting percentages of the adulterants. Therefore, in this study PCA was used as an elementary step before the numeric discriminant analysis. Marchetti et al. made the use of 1H NMR and PLS methods to detect the percentage of pure fruit juices in blends (35); Xu et al. established a non-targeted method using fluorescence quantum dots and one-class PLS for the detection of multiple frauds in orange juice (36). In this study, our PLS discriminant model only requires three important methoxyflavones as variables which makes the detection comparably simpler and more attainable. In addition, the large training and validation sample sizes enable the discriminant model comprehensive and convincible.

#### Detection of Grapefruit Juice Incorporated With Two Categories of Pomelo Juice

The production and consumption of grapefruit juice in China has been increasing rapidly. Grapefruit juice is allowed to be combined with juice obtained from grapefruit hybrids in the United States but no more than 10% by volume (37). However, in China, currently no specific law or standard has been issued to regulate the quality and authenticity of grapefruit juice. Because of the rich resources of pomelos in China and its close flavor and genetic relationship to grapefruit, pomelo juice could hopefully be an ingredient that is legally added to grapefruit juice in the future market, and its addition limit will most likely be under 10%. Therefore, deliberate overblending could be an emerging adulteration.

The vast diversity of pomelos poses a challenge to determine its excess incorporation. As shown in Figure 3, MFs were detected in Shatianyou-related pomelo cultivars PC, PS and PD, and their coumarin/FC profiles were different from those of other cultivars. Therefore, pomelo juices were categorized into two groups, the Shatianyou group (SG) and Wendan group (WG), according to their OHA distributions, and this grouping is generally consistent with the traditional classification results based on botanical traits (38). In this study, 17 grapefruit juices added with 12 SG and 25 WG pomelo juice samples were analyzed using the PLS regression method described above to determine their important OHA variables. The results were largely different between the two combinations, as shown in Table 3. Grapefruit and SG pomelo juices were differentiated by the group of important variables i.e. meranzin (13), 3,5,6,7,8,3',4'-heptamethoxyflavone [3,5,6,7,8,3',4'-HMF, (24)], osthole (31), 6',7'-epoxybergamottin (6',7'-EB, *33*) and bergamottin (36), whereas the difference between grapefruit and WG pomelo juices was characterized by the other group of important variables i.e. bergaptol (6), isomeranzin (14) and 6',7'-dihydroxybergamottin [6',7'-DHB, (25)]. Shatianyou (also spelt as Shatianyu, Shatienyu) and its closely-related cultivars are famous for their highly sweet flavor and low acidity. Their juice pH when fruits mature can be as high as close to 6.0, at which epoxides such as meranzin and 6',7'-EB are stable, as we described previously (39). By contrast, the more acid-stable compounds isomeranzin, 6',7'-DHB and bergaptol were characteristic of the relatively sour juice (pH <4.0) of the WG samples.

**Table 3:** Composition and prediction performance of the two PLS discriminant models to determine the purity of pasteurized grapefruit juice incorporated with the Shatianyou group (SG) and Wendan group (WG) of pasteurized pomelo juices.

		**Added with SG juice**		**Added with WG juice**	
Weighted Regression Coefficient of the important Variables on Factor-1 (98%)		meranzin	−0.450	bergaptol	0.514
		3,5,6,7,8,3',4'-HMF	0.222	isomeranzin	0.486
		osthole	0.288	6',7'-DHB	0.423
		6',7'-EB	0.181		
		bergamottin	0.303		
		Training (Cross validation)	Test (External validation)	Training (Cross validation)	Test (External validation)
Pearson's R^2^		0.974	0.981	0.980	0.793
RMSE (%)		4.115 (RMSECV)	3.543 (RMSEP)	2.733 (RMSECV)	2.590 (RMSEP)
Predicted Purity (PP)	at 100% level	96–103% (*n =* 17)	96–104% (*n =* 7)	97–104% (*n =* 17)	97–104% (*n =* 7)
	at 90% level	87–94% (*n =* 204)	85–94% (*n =* 28)	87–94% (*n =* 425)	87–95% (*n =* 63)
	at 80% level	74–86% (*n =* 204)	76–84% (*n =* 28)	77–85% (*n =* 425)	77–85% (*n =* 63)

*3,5,6,7,8,3',4'-HMF, 3,5,6,7,8,3',4'-heptamethoxyflavone; 6',7'-EB, 6',7'-epoxybergamottin; 6',7'-DHB, 6',7'-dihydroxybergamottin; RMSE, root mean square error (for cross validation, RMSECV; for external validation/prediction, RMSEP)*.

Heated juice samples of grapefruit and two pomelo groups, as shown in Supplementary Table 1, were used to construct the training and external test sets, of which purity had three levels, 100, 90 and 80%. As shown in Table 3, data matrices of both combinations were simplified to one dimension, Factor 1, which explained 98% of the total variance. The Pearson's correlation R^2^ of the two PLS regression models were approximately 0.97 to 0.98, except for that in the external validation of added WG pomelo juices (0.793). Considering that more than half of the WG pomelo juices used for prediction were newly tested cultivars, moderately lower linearity was acceptable. The small values of RMSE (approximate 2.6–4.1%) for cross validation (RMSECV) and prediction (RMSEP) showed that all data in the training set and test set fit well by the two models. The PP range of blended grapefruit juices vs. the real reference value showed a ±3–5% error in each level of the test set.

To sum up, we suggest the following criteria for a conservative assessment of pasteurized grapefruit juice authenticity using the developed OHA models.

1) If the variety or cultivar name of added pomelo juice were presented on label, the corresponding model would apply to assessment; if not, both models should be used to avoid false positive or negative evaluations;
2) If the PP of a grapefruit juice product is no <94% (SG juice incorporation) or 95% (WG juice incorporation), a “grapefruit juice” label that indicates no more than 10% pomelo juice was added can be generally regarded as authentic;
3) If labeled grapefruit juice has a PP < 85% (SG juice incorporation) or PP < 87% (WG juice incorporation), the juice should be determined as fraudulently mislabeled;
4) If the PP value is between 85 and 94% when SG juices are added or between 87 and 95% when WG juices are added, additional methods are required to further verify the authenticity of the juice.

No previous work has been reported investigating authenticity of grapefruit juice when pomelo juice was incorporated as the adulterant. It might because grapefruit is a phylogenic off-spring of pomelo which possesses high genetic diversity. In this study, we categorized the 30 commercial pomelo samples into two groups by carefully analyzing their OHA profiles, and then we were able to construct different discriminant models using a few distinct OHA variables. Jandrić et al. used fingerprinting and targeted metabolomics strategies to study the authenticity of two orange cultivars and a Red blush grapefruit produced in India when their juices were mutual adulterants (40). They found ratios of limonin glucoside to hesperidin, narirutin, and didymin; narirutin to hesperidin and vicenin-2; didymin to hesperidin and narirutin; and vicenin-2 to didymin, have the potential of detecting mutual adulteration down to an impressive level, 2%. However, pomelo juice was not involved and very limited cultivars were studied.

## Conclusions

By using a combination of comparatively inexpensive HPLC separation, diode array detection and fluorescence detection, we identified and quantified 39 oxygenated heterocyclic aglycones (OHAs) and prescreened 13 OHA candidates in citrus juice samples of four varieties, sweet orange, mandarin, grapefruit and pomelo. Characteristic and comprehensive profiles of OHAs in the four juice-species composed of large data from 158 samples in 47 commercial cultivars were drawn for the first time, and chemometric methods were applied to create models for the evaluation of sweet orange and grapefruit juices authenticity. PLS afforded regression models using the least number of the most important OHA variables to determine the proportion of mandarin and pomelo juices added to sweet orange and grapefruit juices, respectively. The discriminant models allowed for a practical, simple and fast preliminary screening of adulteration with a reliable confidence interval, RMSEP < 5%. Considering that juice composition is influenced by a large number of natural and technical factors, such as variety, geographical location, climatic zone, soil, extraction and refining process, further investigations involving worldwide samples and these complex variables are to be expected.

## Data Availability Statement

The datasets presented in this article are not readily available because of the compliance with the National Development Regulations for Protection and Utilization of Agricultural Germplasm Resources of China. Requests to access the datasets should be directed to admin@cric.cn.

## Author Contributions

LH and GL: conceptualization and writing—original draft preparation. LH: methodology. YC, TZ, and GL: validation. LH and QZ: formal analysis. LH, YC, and QZ: investigation. YC, QZ, and WZ: resources. YC and YL: data curation. GL: writing—review and editing, project administration, and funding acquisition. WZ: visualization. TZ: supervision. All authors contributed to the article and approved the submitted version.

## Funding

This work was funded by the Natural Science Foundation of Chongqing, grant number cstc2019jcyj-bshX0104, the Fundamental Research Funds for the Central Universities of China, grant numbers XDJK2020C026 and XDJK2015C017, the National Natural Science Foundation of China, grant number 32101891, the Doctoral Start-up Funding of Southwest University, grant number SWU113100, the Open-project of National Citrus Engineering Research Center of China, grant number NCERC2019004, and the University-local Authorities Cooperation Project of Southwest University, grant number 2020001040.

## Conflict of Interest

TZ is employed by Chengdu Center Testing International Group Co., Ltd. The remaining authors declare that the research was conducted in the absence of any commercial or financial relationships that could be construed as a potential conflict of interest.

## Publisher's Note

All claims expressed in this article are solely those of the authors and do not necessarily represent those of their affiliated organizations, or those of the publisher, the editors and the reviewers. Any product that may be evaluated in this article, or claim that may be made by its manufacturer, is not guaranteed or endorsed by the publisher.
