# Supplementary material for: Targeted Metabolomics With a Chemometric Study of Oxygenated Heterocyclic Aglycones as a Tool for Preliminary Authenticity Assessment of Orange and Grapefruit Juices
Source: Front Nutr. 2022 May 23;9:897982. doi: 10.3389/fnut.2022.897982 (PMC9169518; doi:10.3389/fnut.2022.897982)
Supplement: Supplementary file 1 [file Data_Sheet_1.docx]

Supplementary Material


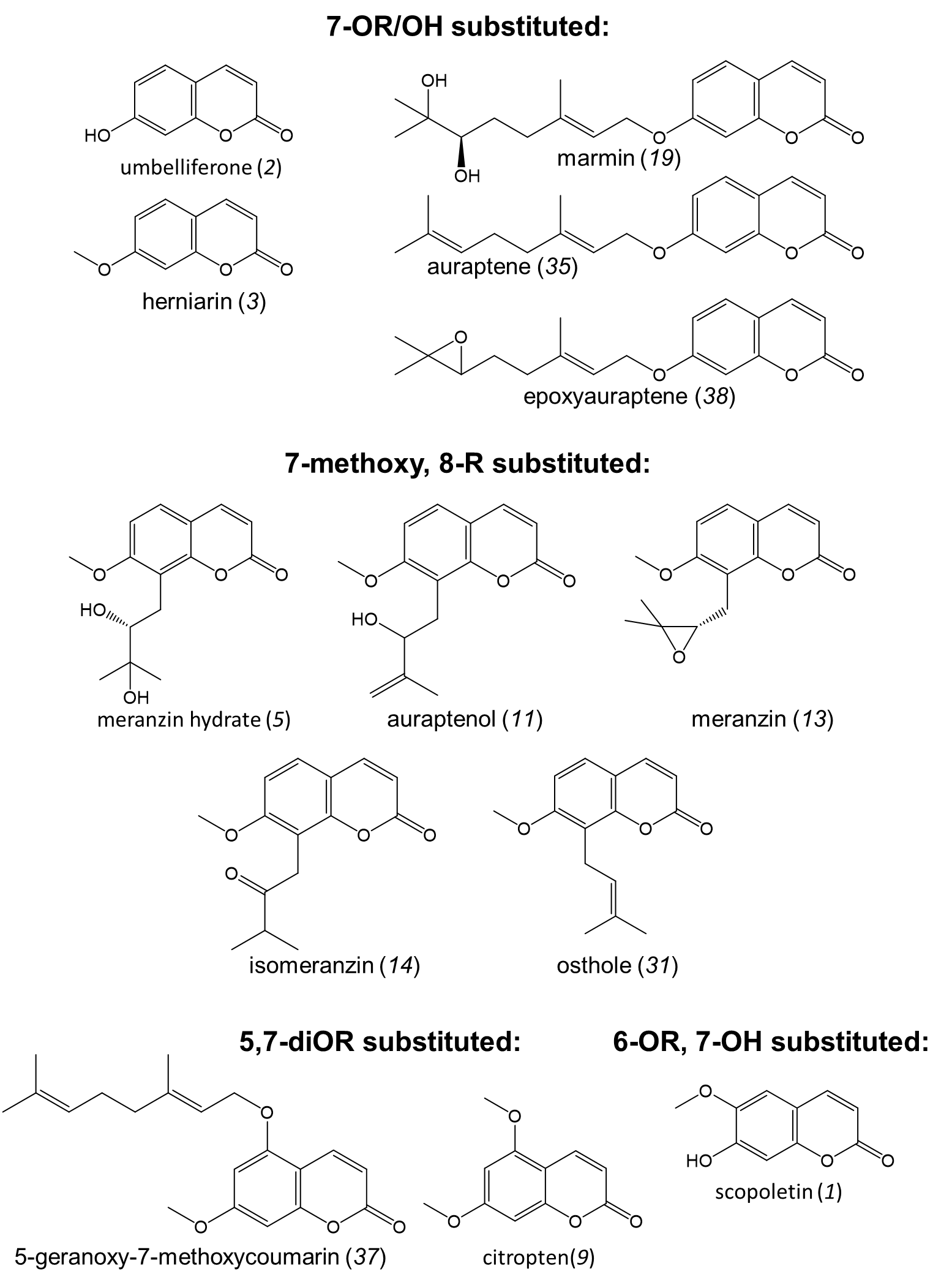


**Supplementary Figure 1.** Molecular structures of the 13 known coumarins grouped by substitution patterns.


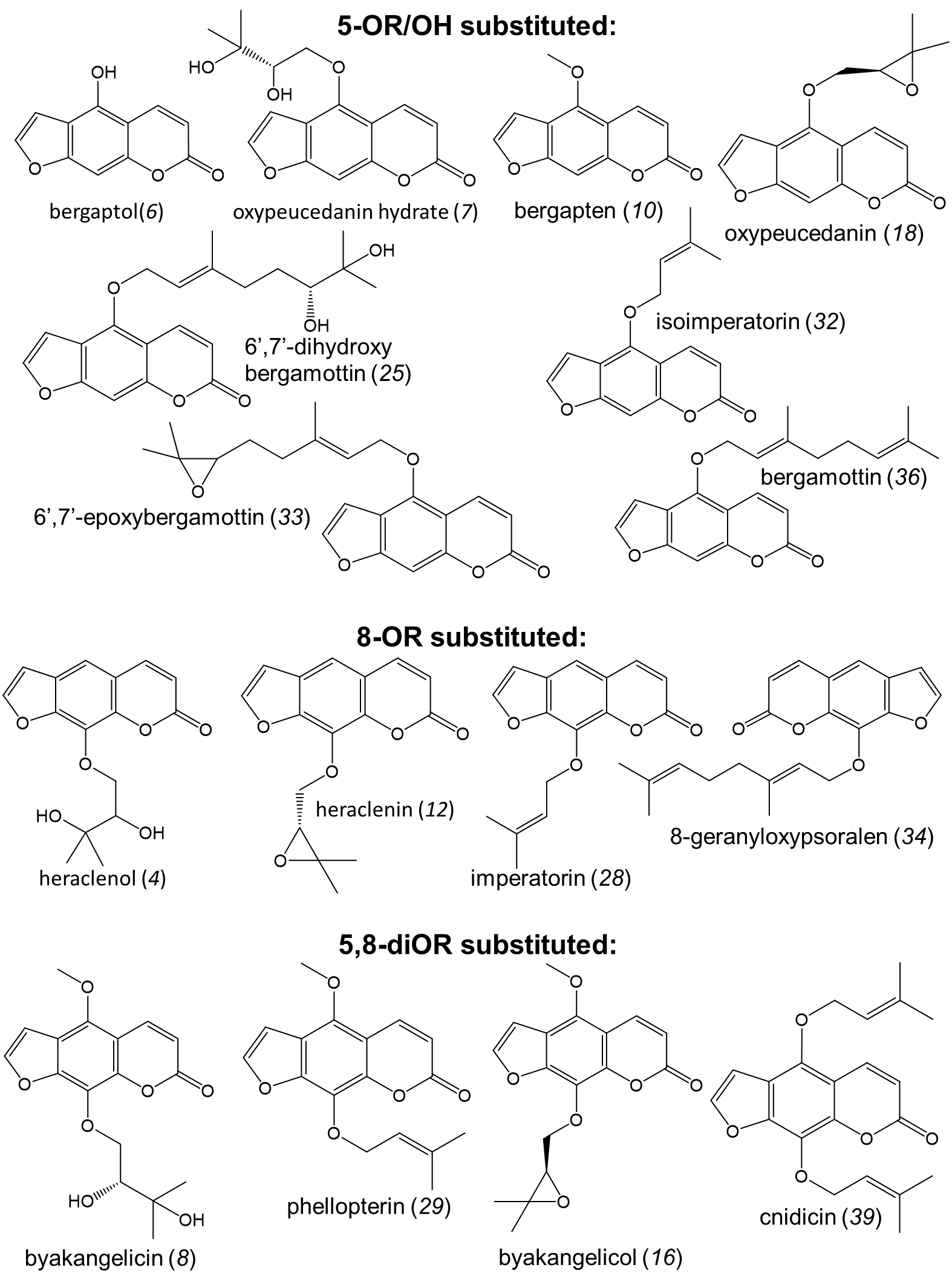


**Supplementary Figure 2.** Molecular structures of the 16 known furanocoumarins grouped by substitution patterns.


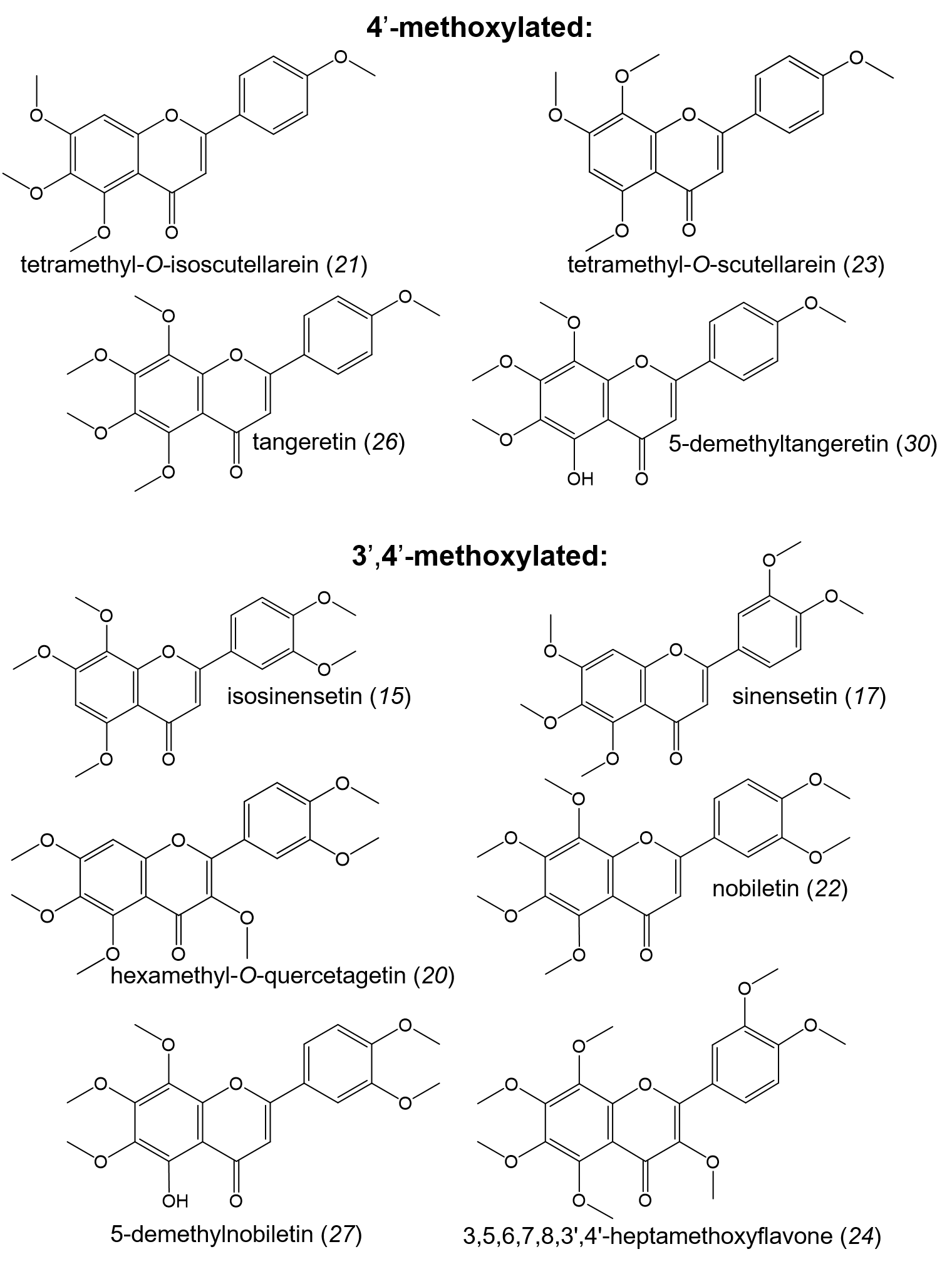


**Supplementary Figure 3.** Molecular structures of the 0 known methoxyflavones grouped by substitution patterns.

**Supplementary Table 1.** Citrus fruit samples obtained for the current study

| **Category** | **Species** | **Cultivar Name** | **Producing Region** | **Abrr. Name** | **Training Sample 1** | **Training Sample 2** | **Training Sample 3** | **Testing Sample** ^a^ |
| --- | --- | --- | --- | --- | --- | --- | --- | --- |
| Sweet orange | *Citrus sinensis* | Valencia | CRI, Beibei, Chongqing (CQ) | OV1 | 2015.6 | 2016.5 | 2017.7 | 2019.6 |
|  |  |  | Jiangyong, Hunan (HN) | OV2 | 2016.4 | 2017.5 | - |  |
|  |  | Olinda Valencia | Laoguancun, Zhongxian, CQ | OO1 | 2015.6 | 2016.4 | 2017.4 | 2019.6 |
|  |  |  | Youyicun, Zhongxian, CQ | OO2 | 2015.6 | 2016.4 | 2017.5 |  |
|  |  |  | Xinlizhen, Zhongxian, CQ | OO3 | 2015.6 | 2016.4 | 2017.5 |  |
|  |  | Delta | Youyicun, Zhongxian, CQ | OD | 2015.6 | 2016.4 | 2017.5 | 2019.6 |
|  |  | Hamlin | Wenbicun, Zhongxian, CQ | OH1 | 2014.12 | 2015.12 | 2016.11 | 2019.1 |
|  |  |  | Wulicun, Zhongxian, CQ | OH2 | 2014.12 | 2016.1 | 2016.12 |  |
|  |  |  | Taiyangcun, Zhongxian, CQ | OH3 | 2015.11 | 2016.1 | 2016.11 |  |
|  |  | Jincheng | Youyicun, Zhongxian, CQ | OJ1 | 2015.2 | 2016.1 | 2017.1 | 2019.1 |
|  |  |  | Fudancun, Zhongxian, CQ | OJ2 | 2015.12 | 2016.12 | 2018.1 |  |
|  |  | Tongshui72-1 | Xinlizhen, Zhongxian, CQ | OTS1 | 2014.12 | 2015.12 | 2016.12 | 2019.1 |
|  |  |  | Taiyangcun, Zhongxian, CQ | OTS2 | 2015.2 | 2016.1 | 2017.1 |  |
|  |  | Changyecheng  early-mature | Diaojiachang, jiangjin, CQ | OCE1 | 2014.11 | 2015.11 | 2016.12 | - |
|  |  |  | Bashanzhen, Zhongxian, CQ | OCE2 | 2014.12 | 2015.12 | 2016.12 |  |
|  |  | Changyecheng  mid-mature | Diaojiachang, jiangjin, CQ | OCM1 | 2015.12 | 2016.12 | 2017.12 | - |
|  |  |  | Shibaozhen, Zhongxian, CQ | OCM2 | 2014.12 | 2015.12 | 2016.12 |  |
|  |  | Changyecheng  late-mature | Diaojiachang, jiangjin, CQ | OCL1 | 2015.1 | 2016.1 | 2017.1 | 2019.1 |
|  |  |  | Xinlizhen, Zhongxian, CQ | OCL2 | 2015.1 | 2016.1 | 2017.2 |  |
|  |  | Zaojin | CRI, Beibei, CQ | OZ | 2014.11 | 2015.11 | 2016.11 | 2019.11 |
|  |  | Yuzaocheng | Jiumangcun, Zhongxian, CQ | OY | 2014.12 | 2015.12 | 2016.11 | - |
|  |  | Trovita | Xinlizhen, Zhongxian, CQ | OT | 2015.2 | 2015.12 | 2016.12 | 2019.1 |
|  |  | Ruby blood orange | CRI, Beibei, CQ | OB | 2016.2 | 2017.1 | 2018.2 | - |
|  |  | Yunguicheng | - | OYG | - | - | - | 2019.1 |
| Mandarin | *C. reticulata* | Xinshengxi No.3 Ponkan | CRI, Beibei, CQ | MPX1 | 2015.12 | 2016.12 | 2017.12 | 2019.1 |
|  |  |  | Changshazhen, Kaizhou, CQ | MPX2 | 2015.12 | 2016.12 | 2017.12 |  |
|  |  | Yanxiwanlu | CRI, Beibei, CQ | MPY | 2016.1 | 2017.01 | 2018.1 | 2019.1 |
|  |  | Bayueju | CRI, Beibei, CQ | MB | 2018.12 | 2019.12 | - | 2019.12 |
|  |  | Nanju | CRI, Beibei, CQ | MN | 2018.11 | 2019.11 | - | 2019.12 |
|  |  | Yongshun Bingtangju | CRI, Beibei, CQ | MYB | 2015.11 | 2016.11 | - | 2019.12 |
|  |  | Caoju | CRI, Beibei, CQ | MC | 2015.12 | 2016.12 | - | 2019.12 |
|  |  | Ugandan Mandarin | CRI, Beibei, CQ | MU | 2017.12 | 2018.12 | - | - |
|  |  | Jinju | CRI, Beibei, CQ | MJ | 2015.11 | 2016.12 | - | 2019.12 |
|  |  | Egypt Mandarin | CRI, Beibei, CQ | ME | 2015.12 | 2016.12 | - | 2019.12 |
|  |  | Nanfengmiju | CRI, Beibei, CQ | MNF | 2015.11 | 2016.11 | 2017.11 | 2019.12 |
|  |  | Shatangju | - | MST | - | - | - | 2019.12 |
|  | *C. tangerina* | Xingyidahongpao | CRI, Beibei, CQ | MXD | 2015.11 | 2016.12 | 2017.12 | - |
|  |  | Roudibahongju | CRI, Beibei, CQ | MRH | 2017.11 | 2018.11 | - | 2019.12 |
|  |  | Chengtuohongju | CRI, Beibei, CQ | MCH | 2016.12 | 2017.11 | - | 2019.12 |
|  |  | Dahongpao | - | MD | - | - | - | 2019.12 |
|  | *C. deliciosa* | Avana N.L. | CRI, Beibei, CQ | MA | 2017.11 | 2018.12 | - | 2019.12 |
|  | *C.nobilis* | Shagan | CRI, Beibei, CQ | MSG | 2015.11 | 2016.12 | - | 2019.12 |
|  | *C. unshiu* | Nangan No.4 Satsuma | CRI, Beibei, CQ | MS | 2015.11 | 2016.12 | 2017.11 | - |
| Grapefruit | *C. paradisi* | Rio Red | CRI, Beibei, CQ | GR | 2015.12 | 2016.12 | 2017.11 | 2020.1 |
|  |  | Thompson | CRI, Beibei, CQ | GT | 2015.12 | 2016.12 | 2017.11 | 2020.1 |
|  |  | Star Ruby | CRI, Beibei, CQ | GS | 2015.11 | 2016.11 | 2017.12 | 2020.1 |
|  |  | Cocktail | CRI, Beibei, CQ | GC | 2017.11 | 2018.11 | 2019.12 | - |
|  |  | Marsh | CRI, Beibei, CQ | GM | 2015.12 | 2016.12 | 2017.11 | 2020.1 |
|  |  | Duncan | - | GD | - | - | - | 2020.1 |
|  |  | Red Marsh | - | GRM | - | - | - | 2020.1 |
|  |  | Flame | - | GF | - | - | - | 2020.1 |
|  | *C. paradise*  *× C.grandis* | Oroblanco | CRI, Beibei, CQ | GO | 2017.11 | 2018.12 | - | - |
| Pomelo | *C. grandis*  (*C. Maxima*) | Changshoushatianyou | Linfengzhen, Changshou, CQ | PC | 2015.11 | 2016.11 | 2017.11 | 2020.1^b^ |
|  |  | Shatianyou | Rongxian, Guangxi | PS | 2015.12 | 2016.12 | 2017.12 | 2020.1^b^ |
|  |  | Dayongjuhuaxin | CRI, Beibei, CQ | PD | 2017.12 | - | - | 2020.1^b^ |
|  |  | Hejiangyou | - | PH | - | - | - | 2020.1 |
|  |  | Guanximiyou | Pinghe, Fujian | PG1 | 2015.11 | 2016.11 | 2016.11 | 2018.11 |
|  |  |  | CRI, Beibei, CQ | PG2 | 2015.10 | 2016.10 | 2017.11 |  |
|  |  | Fengduhongxinyou | Sanyuanzhen, fengdu, CQ | PF1 | 2015.11 | 2016.11 | 2017.11 | 2020.1 |
|  |  |  | CRI, Beibei, CQ | PF2 | 2017.11 | 2018.12 | 2019.12 |  |
|  |  | Anjiangxiangyou | CRI, Beibei, CQ | PA | 2019.12 | - | - | - |
|  |  | Menglunzaoyou | CRI, Beibei, CQ | PM | 2018.9 | - | - | - |
|  |  | Qiyou | CRI, Beibei, CQ | PQ | 2018.10 | 2019.11 | - | - |
|  |  | Wendan | CRI, Beibei, CQ | PW | 2015.10 | 2016.10 | - | - |
|  |  | Zuoshiyou | CRI, Beibei, CQ | PZS | 2018.11 | 2019.12 | - | 2020.1 |
|  |  | Zaoshuyou | CRI, Beibei, CQ | PZ | 2016.10 | 2017.10 | - | - |
|  |  | Naxiyingtaoyou | CRI, Beibei, CQ | PN | 2015.12 | 2016.11 | 2017.11 | - |
|  |  | Dianjiangbaiyou | - | PDJ | - | - | - | 2020.1 |
|  |  | Yuhuanwendan | - | PY | - | - | - | 2020.1 |
|  |  | Wanbaiyou | - | PWB | - | - | - | 2020.1 |
|  |  | Sijipao | - | PSJ | - | - | - | 2020.1 |
|  |  | Liangpingyou | - | PL | - | - | - | 2020.1 |
|  |  | Chandler | - | PCH | - | - | - | 2020.1 |

Notes: ^a^ All test samples were obtained from citrus research institute (CRI) of Southwest University and orchards of Meishan, China, or purchased from local market;^b^ Two batches of samples, one used in training set, the other used for external validation.

**Supplementary Table 2.** Intraday and interday repeatability and recovery of three analyte surrogates using HPLC with PDA detection

| **Surrogate Name** | **Rt** (min) | **Prepared Conc.** (mg/L) |  | **Intraday Repeatability** | | | |  | **Interday Repeatability** | | | |  | **Recovery rate**  (%) |
| --- | --- | --- | --- | --- | --- | --- | --- | --- | --- | --- | --- | --- | --- | --- |
|  |  |  |  | **Rt SD** (min) | **Measured Conc.** (mg/L) | **RSD** (%) | **RE** (%) |  | **Rt SD** (min) | **Measured Conc.** (mg/L) | **RSD** (%) | **RE** (%) |  |  |
| coumarin | 9.3 | 5.0 |  | 0.0 | 5.0 | 2.3 | 0.1 |  | 0.1 | 4.9 | 3.1 | -1.7 |  | 98.2±3.3 |
| xanthotoxin | 13.5 | 5.0 |  | 0.0 | 4.9 | 3.7 | -1.6 |  | 0.2 | 4.9 | 4.5 | -2.0 |  | 101.8±3.9 |
| gardenin A | 41.1 | 5.0 |  | 0.1 | 4.9 | 3.9 | -1.8 |  | 0.4 | 4.9 | 5.9 | -2.1 |  | 98.0±7.5 |
